# Oral health-related knowledge, practice, and utilization of dental services among pregnant women in Riyadh, Saudi Arabia

**DOI:** 10.1371/journal.pone.0319508

**Published:** 2025-04-02

**Authors:** Mamata Hebbal, Eman I. AlSagob, Sree Lalita Kotha, Varkey Nadakkavukaran Santhosh, Ram Surath Kumar, Silvia Mertins, Aalia Alharthi, Nada Aljubran, Shrooq Alqhtani

**Affiliations:** 1 Department of Preventive Dental Sciences, College of Dentistry, Princess Nourah bint Abdulrahman University, Riyadh, Saudi Arabia; 2 Department of Basic Dental Sciences, College of Dentistry, Princess Nourah bint Abdulrahman University, Riyadh, Saudi Arabia; 3 Department of Public Health Dentistry, KLE V.K Institute of Dental Sciences, KLE Academy of Higher Education and Research, Belagavi, Karnataka, India; 4 OBGYN Department, King Abdullah bin Abdulaziz University Hospital, Riyadh, Saudi Arabia; Prince Sattam bin Abdulaziz University, SAUDI ARABIA

## Abstract

**Background:**

Promoting and maintaining appropriate oral hygiene practices in pregnant women is recommended as an integral component of prenatal care to ensure healthy pregnancies and positive birth outcomes. This study aimed to assess the oral health-related knowledge, practices, and utilization of dental services among pregnant women in Riyadh, Saudi Arabia.

**Methods:**

A cross-sectional survey of 1120 pregnant women was conducted in both government and private maternal hospitals from October 2022 to January 2023. A self-administered questionnaire was developed and validated, comprising 37 closed-ended questions. Data analysis involved descriptive analysis, Kruskal-Wallis test, and Spearman correlation coefficient. Multiple linear and multivariable logistic regression analyses were carried out using socio-demographic variables as predictors for knowledge, practices, and utilization of dental services. Level of significance was set at P ≤  0.05.

**Results:**

Majority (37.2%) of the pregnant women were in the 30-34 years age group. The majority had limited oral health knowledge (59.7% very poor, 23.8% poor), and 66.4% had poor oral health practices. Dental service utilization was suboptimal, with 39.6% never seeking dental care during pregnancy. A positive correlation was seen among knowledge, practices, and utilization of dental services. Multiple linear regression revealed that both knowledge and practices scores were significantly associated with education and occupation; and utilization scores with occupation.

**Conclusion:**

This study highlights the need for enhancing oral health knowledge, practices, and utilization of dental services among pregnant women in Riyadh. Bridging knowledge and access gaps can enhance the oral and overall health outcomes of mothers and their infants.

## Introduction

Pregnancy involves a significant physiological process that causes numerous alterations in a woman’s body, impacting both her oral and overall health. Hormonal fluctuations and physiological changes make it a time when oral health disorders are more likely to occur. Oral diseases like periodontitis and dental caries may become more widespread as a result of these changes [1]. Therefore, it is crucial to preserve oral hygiene during pregnancy, considering that the mother’s oral health condition can profoundly impact both her personal well-being and her developing foetus [2].

Dental caries and periodontitis stand out as two of the most prevalent oral health issues on a global scale affecting the general population [3]. Dental caries can lead to pain, infection, and tooth loss if left untreated. Periodontitis, on the other hand, affects the supporting structures of the teeth and can result in gingival inflammation, bleeding, and eventual tooth mobility [4]. These oral health issues significantly impact the general population, placing a substantial burden on both individuals and healthcare systems. Globally, oral health among pregnant mothers was found to be poor. In India, the prevalence of caries and periodontal disease among pregnant women were 62.7% and 95%, respectively [5]. Similarly, a study conducted in Nigeria revealed that pregnant women had a higher prevalence of gingivitis (45.8%) compared to non-pregnant women [6]. A systematic review highlighted that oral health-related quality of life is adversely affected by dental and periodontal problems during pregnancy, with psychological and mental discomfort being the most affected domain [7]. Similar to other countries around the world, Saudi Arabia continues to face the persistent public health challenges of dental caries and periodontitis, both of which require focused attention [8].

The presence of cariogenic bacteria in pregnant mothers can increase the risk of caries development in their infants [9]. Previous research indicates that pregnant women with poor oral health-related quality of life are more likely to have dental caries and periodontal disease, have a poor lifestyle, brush less than twice a day, and be of non-white ethnicity [10]. Furthermore, inadequate oral health during pregnancy might result in negative effects on pregnancy outcomes such as preterm and low birth weight newborns [11]. Therefore, to avoid these potentially harmful effects, it is imperative to maintain oral health throughout pregnancy.

Despite the well-established importance of oral hygiene during pregnancy, many pregnant women worldwide, including those in Riyadh, do not receive adequate oral health care [12,13]. This issue arises from several factors, including a lack of awareness, misconceptions about the necessity of dental care during pregnancy, and limited access to pregnancy-specific dental services [14,15]. The utilization of dental services among pregnant women is crucial for ensuring optimal oral and systemic health during pregnancy. Regular utilization of dental services facilitates early detection and management of oral health diseases, promotes preventive care, and enhances overall well-being [14]. Despite the availability of dental care, utilization rates among pregnant women remain suboptimal, often due to misconceptions about the safety of dental treatments during pregnancy, lack of awareness, and limited access to services [12,13]. Cultural norms and societal beliefs can also play a role in shaping the oral health behaviours among expectant mothers [16]. It is crucial to understand the current gaps in knowledge and oral hygiene practices among expectant mothers to design tailored interventions that improve their oral health outcomes.

Promoting and maintaining good oral hygiene practices among pregnant women should be an integral component of prenatal care to ensure healthy pregnancies and positive birth outcomes. A longitudinal study conducted in India revealed that pregnancy-related oral health education can result in significant improvement in knowledge and attitudes concerning oral health [17]. However, many pregnant women around the world are still unaware of the significance of oral health during pregnancy [12]. While various studies explored the oral health condition of pregnant women worldwide, there has been, limited research focused specifically on the oral health-related knowledge, practices, and utilization of dental services among pregnant women in Riyadh, Saudi Arabia. This knowledge gap underscores the need for targeted studies to better inform healthcare interventions and policies tailored to this population. This study explores the unique demographic and socioeconomic factors that influence oral hygiene behaviours in this specific population. It will provide insights valuable to policymakers and key stakeholders, such as healthcare providers, public health officials, and community leaders, facilitating targeted interventions aimed at enhancing oral health outcomes for pregnant women in Riyadh. By addressing the identified gaps, the findings could also support the development of culturally sensitive educational programs, improved access to dental care services, and the integration of oral health into maternal healthcare policies. The objective of this study is to assess oral health-related knowledge, practices, and utilization of dental services among pregnant women in Riyadh, Saudi Arabia.

## Materials and methods

### Study design and study setting

This study was of cross-sectional design and was conducted among 1120 pregnant women attending government and private maternal hospitals in Riyadh province of Saudi Arabia from October 2022 to January 2023. This study followed the STrengthening the Reporting of OBservational studies in Epidemiology (STROBE) guidelines for observational studies.

### Ethical consideration and informed consent

The study was approved by the Research Ethics Committee at Princess Nourah bint Abdulrahman University (IRB Log No: 22-0453), dated: 02-10-2022. The purpose of the study was explained to all participants and written informed consent was obtained. Those who were willing to give consent were included in the study.

### Sample size and sampling technique

The sample size for this study was estimated using the GPower tool (G*Power statistical software Version 3.1.9.4). With a preset power of 95% and an alpha error of 5%, the sample size was calculated to be 902 [18]. To estimate population parameters, we considered the year 2020 regional statistics in which 2,186,152 females were of reproductive age in Riyadh [19]. To obtain representative results, a sample size of 1120 was chosen. This sample size accounted for approximately 3% of the pregnant women within the total female population aged 15-45 years in Riyadh.

Two-stage random sampling technique was used to select maternity hospitals and pregnant women for data collection in Riyadh. In the first stage, four hospitals were randomly selected from the entire pool (N =  110) of hospitals in Riyadh, ensuring representation from government hospitals, university hospitals, and private hospitals. The four hospitals served as the primary sampling units. In the second stage, pregnant women were randomly selected within each hospital as secondary sampling units. Both stages utilized computer-generated random numbers for random selection.

### Instrument design and pilot testing

The instrument used was a predesigned questionnaire adapted from the study by Malkawi *et al.,* which was modified according to the expert opinion [20]. It comprised of five sections consisting of 37 close-ended questions with multiple-choice options. The first section encompassed the collection of demographic information of the participants which included their age, education, income, and occupation. This was followed by the second section, which focused on the general health status of the pregnant women and multiple responses were allowed for this section. The third section consisting of 16 items primarily addressed oral health-related knowledge, while the fourth section consisting of seven items delved into their oral health practices. The questionnaire culminated with a section pertaining to the utilization of dental services among the participants. It consisted of 10 items for which multiple responses were allowed. The questionnaire distributed was in English as well as in the regional language, Arabic. To verify linguistic validity, the English version was back-translated into Arabic and validated by a language expert. An expert committee performed cross-cultural adaptation on the translated version, with a view of establishing linguistic, idiomatic, experiential, and similarity in concepts involving both the source and target versions [21]. Pregnant women with higher education (graduates, postgraduates, and doctoral) and those proficient in English received the questionnaire in English, while the others received the Arabic version. This method guaranteed that the instrument was appropriate for every participant and that the findings were reliable.

Assessment of the face and content validity of the instrument was carried out by an expert committee consisting of eight subject matter experts. The instrument demonstrated acceptable levels of face validity (86%) and content validity ratio (CVR =  0.80). Test-Retest reliability of the questionnaire was assessed using Kappa statistics. The resulting Cohen’s kappa coefficient was determined to be 0.88, indicating a high level of agreement. Additionally, Cronbach’s alpha coefficient was calculated, yielding a value of 0.85, which demonstrates good internal consistency.

A pilot survey was undertaken involving 50 pregnant women in one of the maternal hospitals in Riyadh. It was conducted to assess the reliability and validity of the questionnaire, the adequacy of participants’ responses, and the feasibility of conducting the investigation. Based on their feedback, the questionnaire was revised further. The results of the pilot study were not included in the main study.

### Data collection, procedure, and response grading

Data was collected utilizing a structured online questionnaire distributed exclusively in the selected hospitals. The participants were presented with a statement explaining the purpose of the study and written informed consent was obtained. The questionnaire completion time was estimated to be approximately 10-15 minutes per individual.

The responses pertaining to the question of oral health knowledge were scored as either 1 or 0 indicating a correct response or an incorrect/I don’t know the response, respectively. The correct response to the questions was summed, and the percentile was determined to assess the overall knowledge of the participants. The knowledge level was categorized as follows: excellent (>75%), good (51%–75%), poor (26%–50%), and very poor (≤25%) [22]. Similarly, response to questions related to oral health practices was scored as 1 or 0 indicating good practice or poor practice, respectively. The overall assessment of oral practice was categorized as good (>50%) or poor (<50%) [22]. Furthermore, questions regarding the utilization of dental services were also scored as 1 or 0 indicating adequate utilization or underutilization, respectively. The overall utilization score was classified as frequent (>50%), occasional (50-1%), and never (0%) [23].

### Data processing and analysis

Data was entered into Microsoft Excel spreadsheets (2019) and analyzed with IBM Corp. 2012, IBM SPSS^®^ Statistics for Windows, Version 21.0. Armonk, NY: IBM Corp. The Shapiro-Wilk test was used to determine the normality of the data distribution, and the data was determined to be not normally distributed. For categorical variables, descriptive statistics were reported as frequencies with percentages, while mean ±  standard deviations were used for continuous variables. Kruskal-Wallis test was employed to determine the difference in knowledge, practice, and utilization of dental services among participants. Spearman’s rank correlation coefficient test along with multivariable analyses such as multiple linear regression and multivariable logistic regression were performed among the various study variables. The level of statistical significance was set at *P* ≤  0.05.

## Results

### Demographic characteristics and health status of pregnant women

A total of 1120 responses (response rate =  100%) were received from pregnant women, with a majority (37.2%) belonging to the age group of 30–34 years. Majority of the women in the present study were homemakers (75.9%) and had graduate level of education (66%). Almost half (44.9%) of the women belonged to the income status of 5000-10000 SR (1 Saudi Riyal =  0.35 US$ [Purchasing Power Parity (PPP) adjusted to United States Dollar (Currency reference year 2024)].) (Refer Table 1).

**Table 1:** Characteristics of the study respondents (*N* =  1120).

Variable Sociodemographic characteristics		*N* = 1120 (%)
Age	<20 years	12 (1.1)
	20–24 years	103 (9.2)
	25–29 years	315 (28.1)
	30–34 years	417 (37.2)
	≥35 years	273 (24.4)
Education	Never attended school	5 (0.4)
	Primary/Middle school	442 (3.9)
	High school	271 (24.2)
	Graduate (Bachelor/Diploma)	739 (66)
	Postgraduate	50 (4.5)
	Doctoral	11 (1)
Income	<5000 SR	120 (10.7)
	5000–10000 SR	503 (44.9)
	10000–15000 SR	314 (28)
	15000–20000 SR	86 (7.7)
	>20000 SR	97 (8.7)
Occupation	Homemaker	850 (75.9)
	Employee full–time	236 (21.1)
	Employee part-time	32 (2.9)
	Retired	2 (0.2)
**Total**		**1120 (100)**

1SR (Saudi Riyal) =  0.27 USD (United States Dollar).

All values are expressed as the frequency with percentages (in parentheses).

More than half of the women (55%) were in their third trimester of pregnancy, whereas 20.9% of them were experiencing their first pregnancy. Majority (99.6%) were non–smokers. More than half (61.9%) of the women were on supplementary medications. They presented with various health conditions like iron deficiency anaemia (34.3%), endocrine disorders (19.6%), urinary tract infections (14.6%), hypotension (10.7%), diabetes mellitus (8.8%) and other conditions. Majority (81.2%) of them utilized governmental healthcare services. However, only 6.6% of the women benefitted from health insurance (Refer Table 2).

**Table 2:** Responses to the health status of pregnant women (*N* =  1120).

Variable		*N* = 1120 (%)
**Health status of pregnant women**		
Term of pregnancy	First trimester	165 (14.7)
	Second trimester	339 (30.3)
	Third trimester	616 (55)
Is this your first pregnancy?	Yes	234 (20.8)
	No	886 (79.1)
If no, how many times have you been pregnant before?	One	241 (21.5)
	Two	207 (18.5)
	Three	162 (14.5)
	Four	110 (9.8)
	Five	91 (8.1)
	More than five	75 (6.7)
Are you a smoker?	Yes	5 (0.4)
	No	1115 (99.6)
If yes, have you stopped smoking during pregnancy?	Yes	4 (0.4)
	No	1 (0.1)
Healthcare provider	Insurance	74 (6.6)
	Self–pay	137 (12.2)
	Governmental	909 (81.2)
**Supplements during pregnancy**		
a. Iron	Yes	893 (79.7)
	No	221 (19.7)
	I don’t Know	6 (0.5)
b. Folic acid	Yes	709 (63.3)
	No	404 (36.1)
	I Don’t Know	7 (0.6)
c. Calcium	Yes	830 (74.1)
	No	279 (24.9)
	I don’t Know	11(1)
d. Progesterone	Yes	89 (7.9)
	No	946 (84.5)
	I don’t Know	85(7.6)
**Conditions during pregnancy** ^¶^		
a. Diabetes mellitus	Yes	98 (8.8)
	No	976 (87.1)
	I don’t Know	46 (4.1)
b. Hypertension	Yes	38 (3.4)
	No	1052 (93.9)
	I don’t Know	30 (2.7)
c. Hypotension	Yes	120 (10.7)
	No	951 (84.9)
	I don’t Know	49 (4.4)
d. Obesity	Yes	64 (5.7)
	No	1009 (90.1)
	I don’t Know	47 (4.2)
e. Bleeding disorders	Yes	30 (2.7)
	No	1058 (94.5)
	I don’t Know	32 (2.9)
f. Iron deficiency anaemia	Yes	384 (34.3)
	No	653 (58.3)
	I don’t Know	83 (7.4)
g. Urinary tract infections	Yes	164 (14.6)
	No	885 (79)
	I don’t Know	70 (6.3)
h. Mental health problems	Yes	49 (4.4)
	No	1049 (93.7)
	I don’t Know	22 (2)
i. Neural health problems	Yes	24 (2.1)
	No	1083 (96.7)
	I don’t Know	13 (1.2)
j. Infectious diseases	Yes	73 (6.5)
	No	996 (88.9)
	I don’t Know	51 (4.6)
k. Endocrine disorders	Yes	220 (19.6)
	No	858 (76.6)
	I don’t Know	42 (3.8)
l. No, I am medically fit	Yes	836 (74.6)
	No	184 (16.4
	I don’t Know	100 (8.9)

¶ Some participants had more than one systematic condition.

All values are expressed as the frequency with percentages (in parentheses).

### Oral health knowledge and practice

Majority of the pregnant women in the present study possessed very poor (59.7%) and poor (23.8%) oral health knowledge. Similarly, majority (66.4%) of the women in this study had poor oral health practices. The findings indicated a statistically significant difference in both knowledge (*P* <  0.001) and practice scores (*P* =  0.004) observed across education, with postgraduates exhibiting the highest median scores for both. Similarly, income distribution also showed significant differences in both knowledge (*P* <  0.001) and practice scores (*P* <  0.001), with higher income groups displaying the highest scores. Occupation–wise distribution showed no significant difference in knowledge (*P* =  0.097). However, the practice scores showed a statistically significant difference (*P* =  0.049), with retired employees having the highest median scores. Table 3 summarizes the participants’ oral health knowledge and practice (S1 File).

**Table 3:** Comparison of demographic characteristics and oral health knowledge/practices/ utilization of dental services scores.

Variable		*n* (%)	Knowledge score			Practice score			Utilization of dental services score		
			Mean ± SD	Median (Range)	*P*–value	Mean ± SD	Median (Range)	*P*–value	Mean ± SD	Median (Range)	*P*–value
**Age**	<20 years	12 (1.1)	2.9 **± ** 2.7	2 (0–8)	0.062	2.8 **± ** 1.2	3 (0–5)	0.171	0.6 **± ** 0.9	0 (0–3)	0.366
	20–24 years	103 (9.2)	4.0 **± ** 3.4	3 (0–16)		2.9 **± ** 1.6	3 (0–6)		1.1 **± ** 1.2	1 (0–6)	
	25–29 years	315 (28.1)	4.5 **± ** 3.7	4 (0–16)		2.7 **± ** 1.5	3 (0–6)		1.1 **± ** 1.4	1 (0–9)	
	30–34 years	417 (37.2)	4.8 **± ** 3.7	4 (0–18)		3.0 **± ** 1.6	3 (0–6)		1.3 **± ** 1.5	1 (0–10)	
	≥35 years	273 (24.4)	4.5 **± ** 3.4	4 (0–15)		2.7 **± ** 1.6	3 (0–6)		1.2 **± ** 1.4	1 (0–9)	
**Education**	Never attended school	5 (0.4)	2.8 **± ** 1.3	3 (1–4)	<.001^**^	2.2 **± ** 0.8	2 (1–3)	0.004^*^	1.0 **± ** 0.7	1 (0–2)	0.119
	Primary/Middle school	442 (3.9)	2.8 **± ** 2.7	2 (0–11)		2.5 **± ** 1.3	3 (0–6)		1.6 **± ** 2.2	1 (0–8)	
	High school	271 (24.2)	3.8 **± ** 3.3	3 (0–16)		2.6 **± ** 1.6	2 (0–6)		1.2 **± ** 1.5	1 (0–10)	
	Graduate (Bachelor/Diploma)	739 (66)	4.8 **± ** 3.6	4 (0–16)		2.9 **± ** 1.6	3 (0–6)		1.1 **± ** 1.3	1 (0–9)	
	Postgraduate	50 (4.5)	7.1 **± ** 4.6	7 (0–18)		3.2 **± ** 1.7	4 (0–6)		1.8 **± ** 1.8	1 (0–6)	
	Doctoral	11 (1)	5.3 **± ** 4.9	3 (0–15)		2.8 **± ** 1.4	3 (1–6)		1.6 **± ** 1.5	1 (0–5)	
**Income**	<5000 SR	120 (10.7)	3.8 **± ** 3.2	3 (0–13)	<.001^**^	2.6 **± ** 1.5	2 (0–6)	<.001^**^	1.4 **± ** 1.6	1 (0–6)	0.059
	5000-10000 SR	503 (44.9)	4.1 **± ** 3.4	3 (0–16)		2.6 **± ** 1.5	3 (0–6)		1.0 **± ** 1.4	1 (0–10)	
	10000-15000 SR	314 (28)	5.0 **± ** 3.7	4 (0–16)		3.0 **± ** 1.6	3 (0–6)		1.2 **± ** 1.4	1 (0–9)	
	15000-20000 SR	86 (7.7)	5.7 **± ** 4.2	5 (0–16)		3.2 **± ** 1.6	3 (0–6)		1.3 **± ** 1.5	1 (0–6)	
	>20000 SR	97 (8.7)	5.5 **± ** 4.0	4 (0–18)		3.3 **± ** 1.6	3 (0–6)		1.3 **± ** 1.5	1 (0–9)	
**Occupation**	Unemployed	850 (75.9)	4.4 **± ** 3.5	4 (0–16)	0.097	2.8 **± ** 1.5	3 (0–6)	0.049^*^	1.1 **± ** 1.4	1 (0–10)	0.008^*^
	Employee full–time	236 (21.1)	5.2 **± ** 4.0	4 (0–18)		3.1 **± ** 1.7	3 (0–6)		1.4 **± ** 1.6	1 (0–9)	
	Employee part-time	32 (2.9)	4.4 **± ** 3.6	4 (0–12)		2.6 **± ** 1.5	3 (0–5)		0.9 **± ** 1.0	1 (0–4)	
	Retired	2 (0.2)	5.0 **± ** 7.1	5 (0–10)		3.5 **± ** 3.5	4 (1–6)		3.0 **± ** 4.2	3 (0–6)	
**Total**		**1120 (100)**	**4.6 ± 3.6**	**4 (0–18)**		**2.8 ± 1.6**	**3 (0–6)**		**1.2 ± 1.5**	**1 (0–10)**	

1SR (Saudi Riyal) =  0.35 US$ [Purchasing Power Parity (PPP) adjusted to United States Dollar (Currency reference year 2024)].

All values are expressed as mean ±  standard deviation (SD) and median with range (in parentheses). The statistical test used: Kruskal Wallis test; level of significance:

** *P* ≤  0.001 is considered highly statistically significant;

* *P* ≤  0.05 is considered statistically significant.

### Utilization of dental services

**Fig 1:**
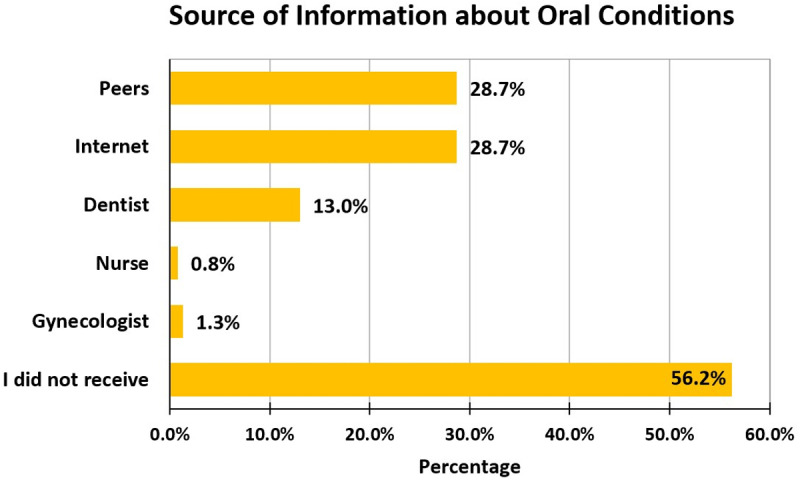
Source of information about oral conditions during pregnancy.

**Fig 2:**
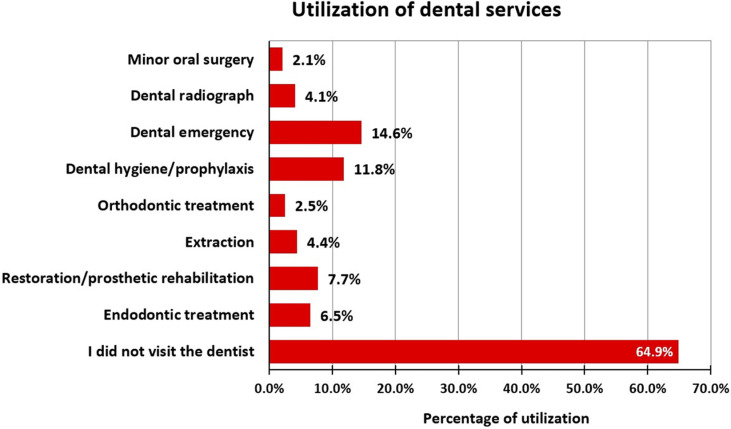
Utilization of dental services behaviour during pregnancy.

The utilization of dental services was found to be below the optimal level, as 39.6% of the women never used dental services during pregnancy, while 58.9% of them occasionally used them. Among the women, only 15.1% received information on oral conditions during pregnancy from healthcare professionals, while the rest received it from the internet and peers. Among healthcare professionals, dentists were the primary source of oral health knowledge for 13% of pregnant women, whereas gynaecologists were the source for only 1.3% of the pregnant women (Fig 1). Majority of the services utilized by these pregnant women were dental emergency (14.6%) followed by dental hygiene/prophylaxis (11.8%) (Fig 2). Occupation–wise distribution showed a statistically significant difference (*P* =  0.008), with highest the utilization of dental services observed among the retired employees (Refer Table 3) (S1 File).

**Fig 3:**
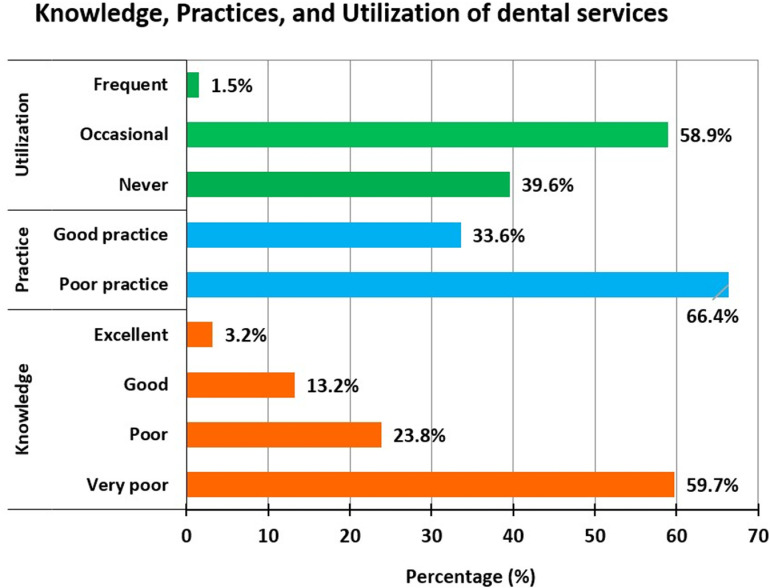
Oral health knowledge, practices, and utilization of dental services among pregnant women.

Fig 3 depicts the oral–health knowledge, practices, and utilization of dental services

### Relationship among knowledge, practices, and utilization of dental services

There was a statistically significant positive correlation observed among knowledge, practices, and utilization of dental services scores (*P* <  0.001). Table 4 presents the field-wise correlation data.

**Table 4:** Correlation between knowledge, practices, and utilization of dental services scores.

Variable	Correlation coefficient (*r*)	*P*–value
Knowledge–Practice	+0.25	<.001^**^
Knowledge– Utilization	+0.27	<.001^**^
Practice– Utilization	+0.25	<.001^**^

The statistical test used: Spearman’s rank correlation coefficient test; level of significance:

** *P* ≤  0.001 is considered a highly statistically significant correlation.

Linear regression model showed a statistically significant relationship between knowledge score with education (β=0.76; 95% CI: 0.47–2.05; *P* < 0.001) and with occupation (β=–0.38; 95% CI: -0.76–0.00; *P* =  0.050). The practice score of the pregnant women also showed a similar relationship with education (β=0.18; 95% CI: 0.06–0.31; *P* = 0.005) and occupation (β=-0.20; 95% CI: -0.36–(-0.03); *P* = 0.019). Meanwhile, the utilization score showed a significant relationship with only occupation of the pregnant women (β=-0.23; 95% CI: -0.38–(-0.07); *P* = 0.004); (Refer Table 5).

**Table 5:** Results of multiple linear regression on factors associated with poor oral health knowledge, practices, and utilization of dental services during pregnancy (*N* =  1120).

Dependent variable		β	SE	*t*	*P*–value	95% CI for β
**Knowledge score**	Constant	1.64	0.62	2.66	0.008^*^	0.43–2.85
	Age (ref: ≥ 35 years)	0.05	0.07	0.80	0.426	-0.08–0.19
	Education (ref: Doctoral)	0.76	0.15	5.14	<.001^**^	0.47–1.05
	Income (ref: > 20000 SR)	0.14	0.11	1.36	0.173	-0.06–0.35
	Occupation (ref: Employee full–time)	-0.38	0.19	-1.96	0.050^*^	-0.76–0.00
**Practice score**	Constant	2.13	0.27	7.88	<.001^**^	1.60–2.66
	Age (ref: ≥ 35 years)	0.05	0.03	1.53	0.126	-0.01–0.10
	Education (ref: Doctoral)	0.18	0.07	2.81	0.005^*^	0.06–0.31
	Income (ref: > 20000 SR)	0.03	0.05	0.71	0.476	-0.06–0.12
	Occupation (ref: Employee full–time)	-0.20	0.08	-2.34	0.019^*^	-0.36–(-0.03)
**Utilization of dental services**	Constant	1.58	0.25	6.28	<.001^**^	1.08–2.07
	Age (ref: ≥ 35 years)	0.03	0.03	0.99	0.323	-0.03–0.08
	Education (ref: Doctoral)	-0.08	0.06	-1.26	0.208	-0.19–0.04
	Income (ref: > 20000 SR)	0.00	0.04	-0.02	0.984	-0.09–0.08
	Occupation (ref: Employee full–time)	-0.23	0.08	-2.86	0.004^*^	-0.38–(-0.07)

The statistical test used: Multiple linear regression analysis model; level of significance: ^**^P ≤  0.001 is considered highly statistically significant; ^*^ *P* ≤  0.05 is considered statistically significant.

β: regression coefficient; SE: standard error; CI: confidence interval.

Logistic regression model (category, aOR, 95% CI, *P*–value) revealed that education (postgraduates, 5.75, 1.11–29.82, 0.037), income [(<5000 SR, 0.46, 0.25–0.85, 0.014); (5000–10000 SR, 0.48, 0.29–0.77, 0.003)] showed statistically significant association with good practice. Similarly, occupation (unemployed, 0.62, 0.44–0.88, 0.008) showed a significant association with frequent utilization of dental services. These results indicated that the pregnant women who possessed higher levels of education, income and occupation had increased odds of having excellent/good knowledge, good practice, and frequent utilization of oral health services (Refer Table 6).

**Table 6:** Results of multivariable logistic regression analysis showing predictors of knowledge, practices, and utilization of dental services (*N* =  1120).

Independent variable		Knowledge^a^ *(Excellent & good vs. Poor & very poor)*		Practice^b^ *(Good vs. Poor)*		Utilization of dental services^c^ *(Never vs. Occasional & frequent utilization)*	
		aOR (95% CI)	*P*–value	aOR (95% CI)	*P*–value	aOR (95% CI)	*P*–value
**Age**	≥35 years	1		1		1	
	20–24 years	0.70 (0.34–1.46)	0.348	1.36 (0.82–2.26)	0.236	1.59 (0.97–2.59)	0.066
	25–29 years	1.31 (0.83–2.07)	0.247	1.08 (0.74–1.56)	0.698	1.07 (0.76–1.50)	0.719
	30–34 years	1.14 (0.74–1.76)	0.550	1.39 (0.99–1.95)	0.059	1.19 (0.86–1.64)	0.296
**Education**	Doctoral	1		1		1	
	Primary/Middle school	0.23 (0.04–1.47)	0.120	2.36 (0.42–13.41)	0.332	0.45 (0.08–2.47)	0.361
	High school	0.46 (0.11–1.95)	0.292	2.65 (0.54–13.13)	0.232	0.49 (0.10–2.40)	0.376
	Graduate (Bachelor/Diploma)	0.65 (0.16–2.61)	0.545	3.53 (0.73–16.94)	0.116	0.47 (0.10–2.24)	0.340
	Postgraduate	1.39 (0.32–6.07)	0.662	5.75 (1.11–29.82)	0.037^*^	0.52 (0.10–2.73)	0.439
**Income**	>20000 SR	1		1		1	
	<5000 SR	0.67 (0.32–1.43)	0.303	0.46 (0.25–0.85)	0.014^*^	0.98 (0.54–1.77)	0.941
	5000 < 10000 SR	0.57 (0.32–1.03)	0.061	0.48 (0.29–0.77)	0.003^*^	0.88 (0.54–1.43)	0.596
	10000 < 15000 SR	0.75 (0.42–1.34)	0.327	0.80 (0.49–1.29)	0.356	1.13 (0.69–1.87)	0.625
	15000 < 20000 SR	1.26 (0.64–2.50)	0.506	1.10 (0.60–1.99)	0.763	0.94 (0.51–1.74)	0.845
**Occupation**	Employee full–time	1		1		1	
	Unemployed	1.20 (0.77–1.85)	0.423	0.96 (0.68–1.35)	0.795	0.62 (0.44–0.88)	0.008^*^
	Employee part-time	1.45 (0.58–3.66)	0.427	0.80 (0.35–1.81)	0.591	0.86 (0.39–1.89)	0.709
	Retired	8.42 (0.41–172.62)	0.167	2.03 (0.11–36.17)	0.630	0.47 (0.03–7.92)	0.604

^a^. reference category: poor & very poor knowledge; ^b^. reference category: poor practice; ^c^. reference category: never- utilization of oral health services.

The statistical test used: Multivariable logistic regression analysis model; level of significance: ^**^P ≤  0.001 is considered highly statistically significant; ^*^P ≤  0.05 is considered statistically significant; Abbreviations: aOR: adjusted odds ratio; CI: confidence interval.

## Discussion

Pregnancy triggers hormonal changes, notably elevated oestrogen, and progesterone levels, crucial for foetal development. These changes enhance gingival blood flow and alter the oral microbiota. Consequently, pregnant women become more vulnerable to gingivitis, an early stage of periodontal disease, which, if untreated, can progress to severe forms [24]. Thus, maintaining appropriate oral hygiene is essential during pregnancy. The present study offers insights into the oral health knowledge, practices, and utilization of dental services among pregnant women in Riyadh.

This study revealed the prevailing deficiency in oral health knowledge among a large number of pregnant women. This knowledge gap can have a significant impact on the oral health of both mother and infant. Pregnant women in Saudi Arabia have varying degrees of knowledge regarding their oral health. According to a study by Gaffar *et al*., more than 70% of pregnant women were aware that pregnancy has a negative impact on oral health [25]. In another study carried out by Asa’ad *et al.,*21% of participants were able to characterize dental plaque, and only 30% were aware of its detrimental effects [26]. In a 22-year comparative survey of the population of the Al-Jubail prenatal clinic, Assery *et al.* found a decline in awareness and oral health among pregnant women [13]. Poor oral health knowledge in pregnant mothers can have several impacts. It can lead to poor oral hygiene practices, which may adversely affect the mother’s oral health and potentially lead to complications such as periodontal disease and dental caries [27,28]. Pregnant women with poor dental health have been linked to lower birth weight newborns [28]. Additionally, low oral health knowledge can result in misconceptions about oral health during pregnancy [29]. Lack of knowledge concerning oral health can also make pregnant women less inclined to seek out dental care during the course of pregnancy [30]. Furthermore, as mothers are largely responsible for establishing their infants’ oral hygiene practices, a lack of awareness about oral health might have an impact on the oral health of the infant [31]. Therefore, improving the knowledge on oral health among pregnant women is crucial for the health of both mothers and infants.

In the present study, it was found that knowledge on oral health among pregnant mothers was influenced by various factors such as age, income, education, and occupation. Wassihun *et al*., in Southern Ethiopia, reported that during the course of pregnancy, having good knowledge on oral health was significantly associated with having access to healthcare, having a higher education, a higher income, and being a government employee [31]. Similarly, a study in the United States found that women with a minimum of an associate’s degree and those with dental health insurance coverage had higher chances of having better oral health knowledge and practices [32]. Hence, among pregnant women, these factors significantly influence the formation of oral health knowledge and practices.

The results of this study also revealed a substantial correlation among the knowledge, practices, and utilization of dental services among pregnant women. In a study by Baskaradoss *et al.,* individuals in Ohio, United States exhibiting lower levels of oral health literacy demonstrated poorer periodontal health. These findings imply that improving oral health literacy may contribute to better overall treatment outcomes and increased adherence to prescribed regimens [33]. Another investigation conducted by Badran *et al.,* in Egypt revealed a notable correlation between oral health literacy and the utilization of dental services [34]. Hence, the level of oral health knowledge during pregnancy exerted a substantial impact on both oral health practices and the utilization of dental services.

In this study, a significant proportion of pregnant mothers were found to have poor oral health practices. These findings were in congruence with a study carried out in Nepal in which 94% of pregnant women acknowledged the need for routine dental care, but only 44% reported dental problems during pregnancy, as oral health was not seen as a priority by 48% of the participants [35]. In Switzerland, the majority of pregnant women sought dental care during their pregnancies; however, it was not done consistently [36]. A study in South India showed that compliance with oral health practices was poor [18].

In this study, a vast majority of the pregnant women utilized oral health services either occasionally (58.9%) or never (39.6%). The study reported that dental emergencies that included dental pain or abscess followed by oral prophylaxis were the most common treatment received among these pregnant women. It was also found that these pregnant women received information on oral conditions from either peers (28.7%) or the internet (28.7%), alarmingly only 2.1% of them received important oral health information from primary healthcare providers during pregnancy that is gynaecologists and nurses. Whereas, dentists provided information to only 13% of the population. These findings were in accordance with those reported by Lazaridi *et al*., in Switzerland [36] and Azarshahri *et al*., in the United States [37]. This indicates that there is a need for dentists and primary healthcare providers such as gynaecologists and nurses to provide more information and oral health education to pregnant mothers.

Similar to the knowledge on oral health, the oral health practices and utilization of dental services were affected by various factors such as age, income, education, and occupation of pregnant women. Amin *et al.,* reported a positive correlation between higher education levels and income with the utilization of oral health services during pregnancy [38]. Moreover, Azarshahri *et al*., reported that pregnant women who held positive dental attitude and were aware of the elevated risk of pregnancy complications associated with poor oral health were more inclined to seek dental care during pregnancy, irrespective of dental coverage [37]. Nevertheless, barriers such as dental care expenses, time constraints, dentists’ reluctance to treat pregnant women, cultural taboos, lack of interprofessional collaboration physiological changes, fear and other psychological conditions were recognized as impediments to the utilization of oral health services during pregnancy, as reported by Bahramian *et al*., in their study conducted in Iran [39].

The demographic characteristics of the surveyed pregnant women indicated a predominant representation in the age group of 30-34 years, characterized as homemakers with a background in higher education. Notably, a significant portion of the respondents were in their third trimester, and they were identified as non-smokers. However, in a study reported by Wahabi *et al*., 31% of pregnant women in Saudi Arabia were subjected to second-hand smoke [40]. In the present study, the majority of pregnant mothers availed healthcare through governmental services, as these services constitute a primary source of healthcare in Saudi Arabia. Notably, these government service providers demonstrated proficiency in detecting high-risk pregnancies [41,42]. The study reveals that a significant majority of pregnant women incorporate pregnancy-related oral supplements like iron, folic acid, and calcium into their regimen. This pattern is particularly pronounced in Saudi Arabia, where the utilization of dietary supplements among pregnant women stands notably high [43]. This study highlights a noteworthy occurrence of iron deficiency anaemia among pregnant mothers. This condition is notably prevalent within the pregnant women demographic in Saudi Arabia. Studies conducted at various healthcare centres provide substantial evidence of this finding [44,45]. Other systemic diseases that were significant in this study included urinary tract infections, endocrine disorders, and hypotension.

Addressing the evident knowledge gap, substandard oral health practices, and limited utilization of dental services becomes not only crucial but obligatory. It necessitates a concerted effort to propagate comprehensive education centred around oral health during the course of pregnancy. Such endeavours are poised to not only preserve the oral health integrity of the mother but crucially contribute to the optimal progress of the developing foetus. The results of this study carry multiple implications for public health interventions and policies directed at enhancing the oral health of pregnant women:

### Oral health promotion

There is an evident necessity for comprehensive oral health education programs specifically designed for pregnant women. These initiatives should emphasize the significance of maintaining optimal oral health during pregnancy and highlight the potential impact on maternal and child health outcomes.

### Healthcare provider involvement

Healthcare professionals, particularly gynaecologists and dentists, can play a pivotal role in disseminating accurate and reliable information about oral health during pregnancy. Integrating discussions on oral health into regular prenatal care visits can play a pivotal role in addressing the information gap.

### Access to oral health services

Efforts should focus on enhancing the accessibility and affordability of dental services for pregnant women. This could involve collaborating with healthcare facilities to ensure that oral health services are available within the same healthcare system and are covered by insurance.

### Tailored interventions

Interventions should be tailored to different socioeconomic groups. For instance, outreach programs targeting lower-income groups and those with lower education levels could focus on addressing barriers to seeking dental care and increasing awareness.

### Community involvement

Peer-to-peer education and community engagement initiatives can be effective in promoting oral health awareness. Engaging community leaders and influencers can help spread effective information within communities.

### Integration with child and maternal health programs

Integrating oral health component into existing child and maternal health programs can enhance overall quality of care provided to pregnant women. This could involve training healthcare professionals to address oral health concerns during prenatal visits.

The present study provides critical insights for policymakers and key stakeholders to address the suboptimal utilization of dental services among pregnant women in Riyadh. By highlighting the gaps in service uptake, the study underscores the need for targeted strategies to improve accessibility and awareness [20]. Policymakers can use these findings to develop evidence-based interventions, such as integrating oral health education into antenatal care programs, ensuring affordable and pregnancy-safe dental services, and implementing community outreach initiatives to dispel misconceptions about dental treatments during pregnancy [38]. Stakeholders, including healthcare providers and public health organizations, can collaborate to train healthcare workers to emphasize the importance of oral health during routine prenatal visits, thereby encouraging higher service utilization [39]. Leveraging the study’s findings, stakeholders can also advocate for policies that prioritize oral health within maternal healthcare frameworks, ultimately fostering better health outcomes for both mothers and their newborns.

The utilization of a cross-sectional design in this study comes with inherent limitations. By relying on questionnaires, there exists a possibility of biases stemming from both recall and socio-desirability factors. Furthermore, due to the nature of the cross-sectional approach, the study cannot establish the temporal relationship of the factors being examined about pregnant women. As it does not allow for determining whether certain oral health knowledge and practices precede or result from pregnancy status. While this research, contributes significantly to our understanding of oral health in pregnant women, these limitations highlight the need for future longitudinal investigations to unravel the temporal intricacies and mitigate potential biases.

## Conclusion

The findings of this study highlights that the pregnant women in Riyadh, Saudi Arabia exhibited very poor knowledge on oral health, engaged in suboptimal practices, and occasionally sought dental services. It emphasizes the requirement for collaborative endeavours among pregnant women in Riyadh to enhance their oral health knowledge, practices, and utilization of dental services. The implications extend beyond oral health, as maternal oral health has a major and extensive influence on both mother and child health. By addressing the gaps in knowledge and access, healthcare systems can contribute to better health outcomes for both mothers and their infants.

## Supporting information

S1 File Supplementary tables. (PDF)
