# Supplementary material for: Oral health-related knowledge, practice, and utilization of dental services among pregnant women in Riyadh, Saudi Arabia
Source: PLoS One. 2025 Apr 2;20(4):e0319508. doi: 10.1371/journal.pone.0319508 (PMC11964211; doi:10.1371/journal.pone.0319508)
Supplement: S1 File — (PDF) [file pone.0319508.s001.pdf]

Responses to knowledge items related to oral health care during pregnancy

**Questions**	**Response**	**Response frequencies *n* (%)**
Do you think there is a need for dental screening during pregnancy?	Yes	532 (47.5)
	No	249 (22.2)
	I don't know	339 (30.3)
What do you think is the appropriate practice after vomiting due to morning sickness?	Eat a light meal, No need to do anything, teeth brushing	627 (56.0)
	I don't Know	91 (8.1)
	Rinse with water	389 (34.7)
	Rinse with sodium bicarbonate and water	13 (1.2)
Do you think there is a relationship between hormonal changes during pregnancy and oral health?	Yes	712 (63.6)
	No	159 (14.2)
	I don't know	249 (22.2)
Do you think there is a relationship between periodontal diseases and pregnancy outcomes?	Yes	512 (45.7)
	No	261 (23.3)
	I don't know	347 (31)
Do you think there is an increased risk of tooth decay during pregnancy?	Yes	574 (51.2)
	No	222 (19.8)
	I don't know	324 (28.9)
Do you think there is an increased risk of tooth erosion during pregnancy?	Yes	468 (41.8)
	No	276 (24.6)
	I don't know	376 (33.6)
Do you think dental treatment is safe during pregnancy?	Yes	385 (34.4)
	No	376 (33.6)
	I don't know	359 (32.1)
Which trimester is the safest to perform treatments?	First trimester	232 (20.7)
	Second trimester	232 (20.7)
	Third trimester	232 (20.7)
	Treatment is safe throughout pregnancy	70 (6.3)
	I don't know	83 (7.4)
**Total**		**1120 (100)**

All values are expressed as the frequency with percentages (in parentheses).

Responses to knowledge items related to oral health care during pregnancy

**Questions**		**Response frequencies *n* (%)**		
		**Yes**	**No**	**I don't know**
What type of dental treatment is safe during pregnancy?	Endodontic treatment	127 (11.3)	147 (13.1)	111 (9.9)
	Restoration/prosthetic rehabilitation	173 (15.4)	114 (10.2)	98 (8.8)
	Extraction	143 (12.8)	143 (12.8)	99 (8.8)
	Orthodontic treatment	181 (16.2)	106 (9.5)	98 (8.8)
	Dental hygiene/prophylaxis	321 (28.7)	29 (2.6)	35 (3.1)
	Dental emergency	302 (27)	45 (4)	38 (3.4)
	Dental radiograph	50 (4.5)	260 (23.2)	75 (6.7)
	Minor oral surgery	135 (12.1)	153 (13.7)	97 (8.7)

All values are expressed as the frequency with percentages (in parentheses).

Practice toward oral health care during pregnancy

**Questions**	**Response**	**Response frequencies *n* (%)**
How often did you visit the dentist prior to pregnancy?	I do not visit the dentist	183 (16.3)
	When I feel pain	627 (56)
	Every 6 months	197 (17.6)
	Every 3 months	113 (10.1)
Did you critically think about the safety of dental treatment options suggested by your dentist when you were not pregnant?	Yes	734 (65.5)
	No	386 (34.5)
How do you describe your commitment to oral hygiene practices prior to pregnancy (ex. frequency of teeth brushing and flossing)?	My oral hygiene practices were better before the pregnancy	459 (41)
	I did not recognize the change	459 (41)
	I do not know	611 (54.6)
	My oral hygiene practices were better during pregnancy	50 (4.5)
Teeth brushing frequency	Not everyday	469 (41.9)
	Once per day	469 (41.9)
	Twice or more per day	651 (58.1)
	I Never brush my teeth	469 (41.9)
Do you floss your teeth?	Yes	533 (47.6)
	No	587 (52.4)
Flossing frequency	Once per day	318 (28.4)
	Once per week	215 (19.2)
	More than once a week	
	Occasionally	
	No	587 (52.4)
Do you use mouthwash?	Yes	560 (50)
	No	560 (50)
**Total**		**1120 (100)**

All values are expressed as the frequency with percentages (in parentheses).

Utilization of dental services during pregnancy

Questions	Response	Response frequencies *n* (%)
How often did you visit the dentist during this current pregnancy?	Once every 3 months	81 (7.2)
	Once every 6 months	57 (5.1)
	When I feel pain	255 (22.8)
	I do not visit the dentist	727 (64.9)
If yes, were you informed about any oral conditions you might experience during pregnancy? ¶	Yes, by my dentist	146 (13)
	Yes, by my gynaecologist	14 (1.3)
	Yes, by my nurse	9 (0.8)
	Yes, by the internet	322 (28.7)
	Yes, by peers	322 (28.7)
	I did not receive any information	629 (56.2)
If you visit a dentist, what was the treatment you received during pregnancy? ¶	Endodontic treatment	73 (6.5)
	Restoration/prosthetic rehabilitation	86 (7.7)
	Extraction	49 (4.4)
	Orthodontic treatment	28 (2.5)
	Dental hygiene/prophylaxis	132 (11.8)
	Dental emergency	164 (14.6)
	Dental radiograph	46 (4.1)
	Minor oral surgery	23 (2.1)
Did you visit the dentist in the six–month period preceding your current pregnancy?	Yes	576 (51.4)
	No	544 (48.6)
If not, please specify the reason. ¶	No time	64 (5.7)
	I do not need	121 (10.8)
	Difficult transportation	54 (4.8)
	Cost	89 (7.9)
	Expecting pregnancy	71 (6.3)
	Dental fear	80 (7.1)
**Total**		**1120 (100)**

¶ Some participants responded with more than one response.
All values are expressed as the frequency with percentages (in parentheses).

Oral health knowledge, practices, and utilization of dental services scores among pregnant women

Variable		*n* (%)
Knowledge	Very poor	669 (59.7)
	Poor	267 (23.8)
	Good	148 (13.2)
	Excellent	36 (3.2)
Practice	Poor practice	744 (66.4)
	Good practice	376 (33.6)
Oral healthcare-seeking behavior	Never	443 (39.6)
	Occasional	660 (58.9)
	Frequent	17 (1.5)
**Total**		**1120 (100)**

All values are expressed as the frequency with percentages (in parentheses).
